# Early Postoperative Results and Complications of using the EX-PRESS Shunt in uncontrolled Uveitic Glaucoma: A Case Series of Preliminary Results

**DOI:** 10.5005/jp-journals-10008-1156

**Published:** 2014-01-16

**Authors:** Jacky WY Lee, Jonathan CH Chan, Li Qing, Jimmy SM Lai

**Affiliations:** Associate Professor, Department of Ophthalmology, Caritas Medical Centre University of Hong Kong, Hong Kong; Associate Professor, Department of Ophthalmology, Queen Mary Hospital, Hong Kong; Clinical Assistant Professor, Department of Ophthalmology, University of Hong Kong Hong Kong; Clinical Professor, Department of Ophthalmology, University of Hong Kong Hong Kong

**Keywords:** EX-PRESS, Uveitic glaucoma, Intraocular pressure, Success, Complications, Medication.

## Abstract

**Purpose:** The purpose of this case series is to describe the efficacy of the EX-PRESS shunt in uveitic glaucoma.

**Methods:** This prospective case series sequentially recruited uveitic glaucoma subjects with intraocular pressure (IOP) > 21 mm Hg despite maximal topical antiglaucoma medications from July 2012 to July 2013 in Hong Kong. All subjects received a trabeculectomy with mitomycin C (MMC) and EX-PRESS shunt implantation. The primary outcome measures included preope-rative IOP and postoperative IOP on day 1, 1 week, 1 month, and every 3 months thereafter. The secondary outcome measures included postoperative complications and follow-up procedures, pre- and postoperative Snellen best corrected visual acuity and cup-disc ratio, as well as the number of antiglaucoma medication required.

**Results:** In a case series of five subjects with uncontrolled uveitic glaucoma, two had inactive anterior uveitis, and three had active panuveitis. The mean preoperative IOP was 35.4 ± 12.6 mm Hg on 3.8 ± 0.5 antiglaucoma eye drops. The mean day 1, 1 week and 1 month IOP's were 6.6 ± 3.7 mm Hg, 7.2 ± 3.2 mm Hg, and 12.6 ± 8.2 mm Hg, respectively. One case required subconjunctival MMC injections postoperatively; two required conjunctival resuture for leakage; and two had early postoperative hypotony that resolved after oral prednisolone. At 6 months, the mean IOP was 13.2 ± 4.6 mm Hg. Four out of five subjects had IOP < 21 mm Hg without medication, and all had IOP < 21 mm Hg with antiglaucoma medication.

**Conclusion:** The EX-PRESS shunt demonstrates good IOP control with a propensity for hypotony in the early postoperative period in this small uveitic glaucoma series.

**How to cite this article:** Lee JWY, Chan JCH, Qing L, Lai JSM. Early Postoperative Results and Complications of using the EXPRESS Shunt in uncontrolled Uveitic Glaucoma: A Case Series of Preliminary Results. J Current Glau Prac 2014;8(1):20-24.

## INTRODUCTION

Glaucoma is a serious complication that can occur in 10 to 20% of uveitis and even a higher prevalence in cases of Fuchs heterochromic cyclitis, herpes, or sarcoidosis-related uveitis.^1-3^ The intraocular pressure (IOP) elevation in uveitic glaucoma can be due to a number of reasons including: increased aqueous viscosity and protein,^4,5^ reduced trabecular meshwork function by infammatory cytokines, pigments, trabeculitis and eventual trabeculocyte cytotoxicity.^6^ In addition, the treatment of uveitis with topical corticosteroids can also induce significant IOP ele vation. Around 21% of adults have been reported to have intermediate IOP rise after topical dexamethasone use.^7^

The treatment of IOP rise in uveitis is most commonly with topical or oral antiglaucoma medications, but the response to treatment is often variable ranging from 0 to 80% reductions. When maximal antiglaucoma medications fail to control IOP, fltration surgery is often required; although, the success rate in uveitic glaucoma is often poorer than that of primary glaucomas. The success rate of trabecu lectomy in uveitic glaucoma, without the use of adjunc tive anti metabolites, is only about 30% and slightly improved to 50% at 5 years with the use of 5-fluorouracil (5-FU).^8^

The EX-PRESS glaucoma fltration device is a metallic implant that provides an artificial channel to drain the aqueous into the subconjunctival space and it is less invasive and more precise than the traditional trabecu lectomy. It provides a significantly lower IOP in the first 3 years postoperatively and less antiglaucoma medication requirements 5 years postoperatively compared to trabecu lectomy.^9^ The EXPRESS does not require a sclerectomy or peripheral iridec-tomy; hence, there is less infammation and less risk of blockage of the inner window by fibrin, blood, or iris. The rate of postoperative hypotony (4% *vs* 32%) and choroidal effusion (8% *vs* 38%) is also significantly less with the EXPRESS than in traditional trabeculectomy.^10^

While, the efficacy of the EX-PRESS has been established in the use of primary open angle glaucoma (POAG), its efficacy in uveitic glaucoma is less well studied. The purpose of this case series was to describe the efficacy of the EX-PRESS mini shunt device in uncontrolled uveitic glaucoma.

## PATIENTS AND METHODS

This prospective case series adhered to the tenets of the Declaration of Helsinki. Informed patient consent and approval by the Institutional Review Board were obtained prior to study commencement. The authors declare no financial or proprietary interests. All subjects were recruited sequentially from the ophthalmology clinic of a tertiary university hospital, Queen Mary Hospital, in Hong Kong during July 2012 to July 2013. The inclusion criteria consis ted of consenting individuals with uncontrolled uveitic glau coma with IOP > 21 mm Hg despite maximal topical anti glaucoma medications. The exclusion criteria included: age < 18 years, those who were unable to consent, only eye, previous glaucoma surgery, or those with less than 6 months follow-up. The surgical procedure for EX-PRESS shunt implantation was as follows:

1. Local anesthesia with Xylocaine gel 2% (AstraZeneca, 1004 Middlegate Road, Ontario, Canada).
2. Fornix-based conjunctiva opening.
3. Application of mitomycin C (MMC) 0.4 mg/ml for 3 minutes followed by irrigation with balanced salt solution.
4. A 4 × 4 mm partial thickness sclera fap.
5. Paracentesis and anterior chamber reformation with a cohesive viscoelastic.
6. Injection of the EX-PRESS shunt (50 mm) into the ‘blue-line' junction adjacent to the clear cornea with the aid of a 27-gauge needle.
7. No sclerectomy and peripheral iridectomy was required.
8. The sclera fap was closed with 10-O Nylon.
9. The conjunctival wound was closed with 10-O Nylon or 8-O Vircyl in an interrupted suture manner.

The primary outcome measures were preoperative IOP and postoperative IOP on day 1, 1 week, 1 month and every 3 months thereafter. The secondary outcome mea sures included postoperative complications and follow-up procedures, pre- and postoperative Snellen best corrected visual acuity (BCVA), and cup-disc ratio (CDR), as well as the number of antiglaucoma medication required.

Descriptive analysis of IOP, number of complications and procedures, pre- and postoperative eye drops, and BCVA and CDR were presented. Means were expressed as mean ± standard deviation. Complete success was defined as IOP ≤ 21 mm Hg without antiglaucoma medication and partial success was defined as IOP ≤ 21 mm Hg with topical antiglaucoma medication.

## RESULTS

There were five patients recruited during the study period. All subjects were males, ethnic Chinese with a mean age of 48.4 ± 12.9 years. There were three right eyes and two left eyes. All subjects had uncontrolled uveitic glaucoma with IOP > 21 mm Hg despite maximum topical antiglaucoma eye drops and open angles on gonioscopy. The mean preoperative CDR was 0.6 ± 0.3.

Two subjects had anterior uveitis and had no ocular infa mmation for 6 months prior to the EX-PRESS implantation. Three subjects with panuveitis had persistent infam-mation leading up to the EX-PRESS implantation and were on oral steroids or oral immunosuppressants (cyclosporine and azathioprine). Two of these three patients had a posterior vitrec tomy to clear up the vitritis and one required repeated intravitreal steroid injections prior the EX-PRESS implantation.

**Graph 1:**
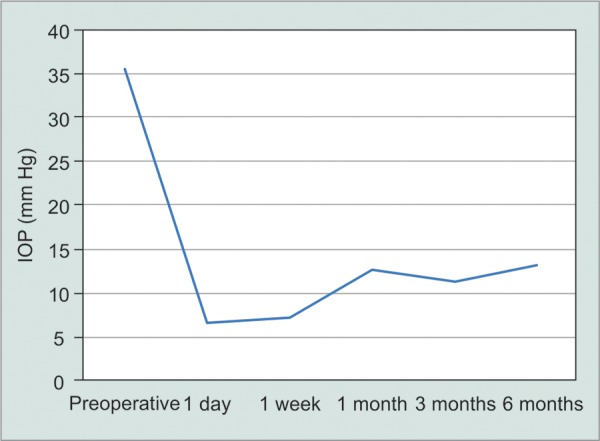
IOP change after EX-PRESS in uveitic glaucoma

The preoperative Snellen BCVA ranged from 0.1 to counting fingers (CFs). The mean number of preoperative antiglaucoma eye drops was 3.8 ± 0.5 and the three subjects with active panuveitis required oral acetazolamide 250 mg four times daily. The mean preoperative IOP was 35.4 ± 12.6 mm Hg with maximal medication. The mean day 1, 1 week, and 1 month IOP's were 6.6 ± 3.7 mm Hg, 7.2 ± 3.2 mm Hg, and 12.6 ± 8.2 mm Hg respectively (Graph 1).

Postoperatively, one case required four subconjunctival MMC (0.02 mg in 0.1 ml) injections during the first month for early postoperative scarring as determined by prominent conjunctival vascular injection and a fattened bleb. Two cases required conjunctival resuture for conjunctival recession with leakage postoperatively. Two cases had early postoperative hypotony due to ciliary body shutdown that resolved within the first month after oral prednisolone of 30 mg daily (0.5 mg/kg/day) for 1 week with gradual tapering. Of these two cases, one developed a shallow anterior chamber with near iridocorneal touch requiring anterior chamber reformation with viscoelastics. This case was further complicated by choroidal detachment with residual hypotony maculopathy with a 6-month post operative BCVA of 0.1 (preoperative BCVA was CF) (Table 1).

At 3 months, the mean IOP was 11.4 ± 3.6 mm Hg. At 6 months, the mean IOP was 13.2 ± 4.6 mm Hg and the mean number of antiglaucoma medication was 0.8 ± 1.8. Four out of the five subjects had IOP < 21 mm Hg at 6 months without antiglaucoma medication (complete success) and all had IOP < 21 mm Hg with antiglaucoma medication (partial success). The mean BCVA and CDR at 6 months were 0.4 ± 0.3 and 0.6 ± 0.3, respectively.

**Table 1:** **Table 1:** Summary of early postoperative complications and results for EX-PRESS in uveitic glaucoma

*Subject demographics*										*Early postoperative complications*												*Anti-glaucoma eye drops*				*Cup-disk-ratio*				*Snellen best corrected VA*			
*Case No.*		*Sex*		*Age*		*Operated eye*		*Diagnosis*		*Early hypotony (ciliary body shutdown)*		*Choroidal effusion*		*Shallow anterior chamber*		*Hypo-tonous maculo-pathy*		*Bleb leak (conjuctival recession*		*Bleb scaring*		*Preoperative eye drops (no.)*		*6 mth eye drops (no.)*		*Preoperative*		*6 mths*		*Preoperative*		*6 mths*	
1		M		62		Right		Panuveities (active)		–		–		–		–		–		–		3		0		0.6		0.4		0.1		0.5	
2		M		49		Right		Anterior uveitis (inactive)		–		–		1		–		1		1		4		4		0.9		0.9		0.3		0.4	
3		M		52		Left		Anterior uveitis (inactive)		–		–		–		–		1		–		4		0		0.9		0.9		0.7		0.8	
4		M		52		Left		Panuveitis (active)		1		1		–		1		–		–		4		0		0.4		0.4		CF		0.1	
5		M		27		Right		Panuveitis (active)		1		–		1		–		–		–		4		0		0.4		0.4		CF		0.05	

## DISCUSSION

The management of glaucoma in uveitis poses considerable challenges. The success rate of using glaucoma drainage device in uveitic glaucoma is higher than in standard trabe-culectomy. The success rate for the Molteno drainage device has been reported to be 87% at 5 years,^11^ 94% at 1 year for the Ahmed drainage device,^12^ and 91.7% at 2 years for the Baerveldt drainage device.^13^ In our small case series, the complete success of EX-PRESS shunt for uveitic glaucoma is 80% (4/5) at 6 months without medication, and the partial success is 100% (5/5) at 6 months with antiglaucoma medication. Our findings are in line with a study by Reyes et al,^14^ reporting a success of 86% with or without medication and 73% without medication at 10 months after EX-PRESS. However, the population in Reyes' study consisted of both primary and secondary glaucomas, including uveitic glaucoma, thus, their success is likely to be higher than for uveitic glaucoma alone.

The use of postoperative 5-FU injections was demonstrated to improve the success of trabeculectomy in uveitic glaucoma by 50%.^8^ However, given the predisposition to ciliary body toxicity from the absorption of antimetabolites, the risk and benefit of which must be balanced. In our case series, one subject with signs of early bleb failure, including conjunctival injection and a fattened bleb, received four postoperative injections of subconjunctival MMC within the first month. The bleb was salvaged and he achieved a complete success at 6 months. As for the choice of MMC *vs* 5-FU, reports have shown similar efficacy between the two in terms of IOP control.^15-17^ The use of postoperative antimetabolite injections was avoided in our subjects with overfltration and ciliary body shutdown.

Postoperative hypotony is common in uveitic glaucoma because uveitis itself releases infammatory cytokines that are toxic to the ciliary body,^18^ and the use of intraoperative MMC can further exacerbate ciliary body toxicity. A history of posterior vitrectomy for vitritis also predisposes the eye to hypotony due to removal of the supporting vitreous. In our series, two cases had ciliary body shutdown with hypotony but both resolved within the first month after a short course of oral steroids. For the case that had a resultant hypotony maculopathy, he had a posterior vitrectomy within 1 month prior to the EX-PRESS implantation; his BCVA after the EX-PRESS procedure improved from his preoperative level. Kaburaki et al^19^ reported that the rate of hypotony following trabeculectomy for uveitic glaucoma was 28.3%; however, this rate was for long-standing ocular hypotony in a population of inactive uveitis at the time of surgery. Thus, a direct comparison cannot be made with our series that reports early hypotony.

In theory, it is best to wait until the intraocular infamma-tion is settled for months before glaucoma surgery for uveitic glaucoma but in reality, the IOP is commonly uncontrolled during active infammation and like in our series, three of the subjects had active panuveitis with uncontrolled IOP despite maximal topical and systemic anti glaucoma medications. Des pite implanting the EX-PRESS in the setting of active uveitis, all three subjects were able to achieve complete success at 6 months.

The EX-PRESS shunt has been advocated to offer better early postoperative IOP stability and less hypotony due to a smaller lumen size (50 or 200 mm) versus the largely visible sclera window of a traditional trabeculectomy. The published early postoperative hypotony rates for EX-PRESS in POAG ranges from 4.0 to 47.2% *vs* 14.0 to 47.4% in traditional trabeculectomy.^20^ The rate of postoperative hypotony in uveitic glaucoma is expected to be higher like in our series, 40% (2/5), due to the possibility of ciliary body shutdown from both infammation and antimetabolite toxicity as well as after posterior vitrectomy for vitritis. Compared to the Ahmed valve that has a resistance to aqueous outfow, the rate of early postoperative hypotony is around 9% in those with uveitis from systemic disease.^21^ However, glaucoma drainage devices are associated with other complications like corneal decompensation, extraocular motility disorders, conjunctival thinning, and hypertensive phase that are seldom seen with the EX-PRESS. Cyclophotocoagulation is an alternative treatment modality but has a postoperative hypotony rate of 19% in uveitic glaucoma^22^ together with risks of increased infammation, retinal detachment and phthisical changes.

Very few studies have reported the early postoperative results and complications of using the EX-PRESS shunt for uveitic glaucoma alone. The surgical approach to the management of uveitic glaucoma will vary depending on center and surgeon preference. Our single center, non comparative case series has reported the early postoperative outcomes and complications of using the EX-PRESS in a small sample of uveitic glaucomas. The EX-PRESS shunt can successfully normalize IOP in the majority of uveitic glaucoma subjects, although, there is a higher propensity for hypotony in the early postoperative period compared to other glaucoma drainage devices. Larger trials are warranted to establish the long-term efficacy of using the EX-PRESS for the treatment of uveitic glaucoma, as well as to compare its efficacy with traditional trabeculectomy and glaucoma drainage devices.
